# *Notes from the Field:* Support for Wastewater Monitoring and Influence on Protective Behavioral Intentions Among Adults — United States, July 2024

**DOI:** 10.15585/mmwr.mm7337a2

**Published:** 2024-09-19

**Authors:** Rieza H. Soelaeman, Danielle Kleven, Jena Losch, Michael Vega, S. Nicole Fehrenbach, Jessica N. Ricaldi, Diana Valencia, Scott Santibañez

**Affiliations:** ^1^Division of Infectious Disease Readiness and Innovation, National Center for Emerging and Zoonotic Infectious Diseases, CDC; ^2^Association of Schools and Programs of Public Health, Washington, DC; ^3^Office of the Director, National Center for Emerging and Zoonotic Infectious Diseases, CDC.

**Summary**

**What is already known about this topic?**

Wastewater monitoring has expanded since 2020, providing data for several infectious diseases.

**What is added by this report?**

In a survey of public support, U.S. adult residents (74.6%) strongly or somewhat support wastewater monitoring, with nearly all (95.3%) stating they would take steps to protect themselves if wastewater monitoring data indicated disease transmission in their area.

**What are the implications for public health practice?**

Making infectious disease wastewater data readily available helps keep the public informed and can facilitate early adoption of protective health behaviors. Presentation of these data should be accompanied by clear public health interpretations.

In 2020, during the COVID-19 pandemic, CDC established the National Wastewater Surveillance System and later expanded it to include mpox and influenza A data dashboards.^†^ Wastewater utility partners have cited community health benefits as a motivating factor for participating in wastewater surveillance; a lack of public support for wastewater surveillance activities might lead utility partners to cease participation (*1*,*2*). However, little is known about public support for wastewater monitoring and its influence on protective health behaviors. As innovative surveillance strategies such as wastewater surveillance evolve, ethical considerations, including understanding public perceptions regarding support for these activities and potential risks to communities, are essential (*3*).

## Investigation and Outcomes

During July 24–26, 2024, Porter Novelli Public Services^§^ conducted a nationwide nine-question survey in English, developed with input from CDC, among U.S. adults regarding support for wastewater monitoring of infectious diseases and protective health behavior intentions, to guide public messaging about wastewater surveillance. This activity was reviewed by CDC, deemed not research, and was conducted consistent with applicable federal law and CDC policy.**^¶^** Nonprobability quota sampling was used to select 1,016 respondents. The sample was weighted by gender, age, region, race and ethnicity, and education to match the U.S. population composition using Current Population Survey proportions.**

Data from this survey were analyzed for 1) overall support for wastewater monitoring of infectious diseases, 2) support for access to wastewater data regardless of known interpretation of risk to the public, 3) protective health behaviors respondents would take if wastewater data indicated that a virus such as influenza were spreading in their area, and 4) differences in support and protective behavioral intentions by sociodemographic factors. Responses were analyzed by respondent characteristics. Statistical significance was determined at α = 0.05 using Pearson chi-square tests corrected for survey design. Analyses were conducted using Stata (version 17.0, StataCorp).

## Overall Support for Wastewater Surveillance for Infectious Diseases

Four survey items on support for wastewater monitoring of specific types of pathogens with a Cronbach’s α of 0.91 were averaged into a single measure of overall support. Almost three quarters of respondents (74.6%) strongly or somewhat supported public health department monitoring of wastewater for infectious diseases (Table). Support for wastewater monitoring was similar among persons of different races and ethnicities (p>0.9) but differed significantly by age, education, and marital status.

**TABLE:** Levels of support for wastewater monitoring and behavioral intentions among adults — Porter Novelli View survey, United States, July 2024*

Characteristic	No. (%)^§^	Weighted row %									
		Level of support for public health departments monitoring wastewater for infectious diseases^†^				Would like access to wastewater data even when there is not enough information to determine public health risk or protective behaviors				Would use one or more protective behavior if wastewater data showed that a virus such as influenza were spreading in area	
		Strongly or somewhat support	Neutral	Strongly or somewhat oppose	p-value^¶^	Strongly or somewhat agree	Neutral	Strongly or somewhat disagree	p-value^¶^	Yes	p-value^¶^
**Total**	**1,016 (100.0)**	**74.6**	**17.9**	**7.5**	—	**57.8**	**26.4**	**15.8**	—	**95.3**	—
**Gender identity**											
Female	506 (50.9)	76.0	16.8	7.2	0.18	53.5	28.8	17.8	0.04	96.0	0.40
Male	500 (48.6)	73.5	18.7	7.8		62.3	23.9	13.8		94.6	
Other	10 (0.5)	47.7	52.3	0.0		58.4	33.6	8.1		100.0	
Race and ethnicity**											
Black or African American	118 (12.1)	71.4	19.0	9.6	0.93	67.5	21.4	11.1	0.01	96.8	0.07
White	640 (61.3)	75.7	17.5	6.8		52.9	28.0	19.1		93.9	
Hispanic or Latino	143 (17.5)	72.5	19.2	8.3		64.2	26.1	9.7		97.9	
Other race^††^	115 (9.1)	75.4	16.8	7.8		65.5	23.0	11.6		98.2	
**Age group, yrs**											
18–29	220 (19.8)	62.0	24.8	13.2	<0.001	61.9	25.0	13.2	0.32	96.2	0.75
30–39	197 (18.4)	70.6	20.5	9.0		61.2	27.4	11.4		95.9	
40–49	154 (15.0)	78.8	15.4	5.8		61.2	23.1	15.7		96.5	
50–64	246 (24.4)	77.9	16.5	5.6		55.0	26.4	18.7		94.4	
≥65	199 (22.4)	82.8	12.9	4.3		52.2	29.2	18.7		94.3	
**Employment status**											
Employed	586 (54.1)	73.8	18.6	7.5	0.80	65.1	21.9	13.0	<0.001	97.5	<0.001
Not employed	430 (45.9)	75.5	17.0	7.4		49.2	31.7	19.1		92.8	
**Education**											
High school or less	327 (38.9)	71.3	19.3	9.4	0.05	59.7	25.9	14.4	0.67	94.5	0.30
Some college	264 (24.7)	71.0	21.2	7.7		55.9	28.5	15.6		94.5	
Bachelor’s degree	251 (22.1)	80.9	14.5	4.6		57.2	27.3	15.5		97.5	
Any postgraduate education	174 (14.3)	80.3	13.6	6.2		56.8	22.8	20.4		95.7	
**Marital status**											
Currently married or in a union	529 (50.1)	78.1	16.1	5.8	0.03	55.7	27.3	17.0	0.53	95.9	0.66
Divorced, widowed, or separated	182 (19.9)	71.7	22.0	6.3		56.7	28.2	15.1		94.5	
Never married	305 (30.0)	70.7	18.2	11.1		62.0	23.7	14.3		94.9	
**Community type**											
Rural	218 (22.3)	71.5	23.1	5.4	0.08	51.0	27.4	21.6	0.04	94.5	0.71
Suburban	508 (48.9)	76.6	16.5	6.9		58.0	26.2	15.7		95.3	
Urban	290 (28.8)	73.6	16.3	10.0		62.6	25.9	11.5		96.1	

* Survey was administered in English online during July 24–26, 2024.

^†^ Levels of support for wastewater monitoring were similar across four separate pathogen categories (Cronbach's α = 0.91); therefore, categories were averaged into a single measure of overall support.

^§^ Unweighted counts and weighted column percentages.

^¶^ Statistical significance of differences in responses was determined at α = 0.05 using Pearson chi-square tests corrected for survey design.

** Persons of Hispanic or Latino (Hispanic) origin might be of any race but are categorized as Hispanic; all racial group tabulation is limited to those persons reporting being non-Hispanic.

^††^ Includes American Indian or Alaska Native, Asian or Asian-American, Middle Eastern or North African, and Native Hawaiian or Pacific Islander persons, and persons identifying as more than one race.

## Support for Data Availability Regardless of Known Public Health Risk or Protective Behaviors

Respondents strongly or somewhat agreed (57.8%) that they wanted access to rapid wastewater data, even if information to determine public health risk or specific protective actions is insufficient (Table). The percentages of persons who indicated that they would like to see rapid wastewater data were higher among non-Hispanic Black or African American persons (67.5%), Hispanic or Latino (Hispanic) persons (64.2%), and non-Hispanic persons from other racial groups (65.5%) than among non-Hispanic White persons (52.9%) (overall p<0.01). Those most supportive of rapid access to wastewater data included men (p<0.05), persons who were employed (p<0.001), and residents of urban or suburban communities (p<0.05).

## Intention for Data-Informed Protective Behaviors

Almost all respondents (95.3%) would consider at least one protective health behavior if wastewater data indicated a virus such as influenza in their area. Behaviors most likely to be considered included more frequent handwashing (76.1%), avoiding large gatherings (61.1%), and avoiding visiting persons at higher risk for infection-related complications (59.1%) (Supplementary Table, https://stacks.cdc.gov/view/cdc/162074).

## Preliminary Conclusions and Actions

The findings in this report are subject to at least five limitations. First, because this survey used an Internet panel, persons with limited Internet access or technological proficiency might not have been able to participate. Second, public awareness of wastewater surveillance might vary geographically, and participation might have been higher among persons with higher levels of awareness than the average U.S. resident. Third, responses might be subject to social desirability bias, or the tendency of respondents to report what they believe is desirable, rather than their true opinions or behaviors (*4*). Fourth, this survey was intended to gauge public support for wastewater monitoring of infectious diseases; public support for other uses of wastewater monitoring might differ from what is reported here. Finally, because the survey was administered in English only, these data do not include the perceptions of persons with limited English proficiency.

These findings indicate strong support for wastewater monitoring for infectious diseases among U.S. adults across various sociodemographic groups and intention to use reported wastewater data to guide certain health-related behaviors. In addition, most respondents indicated that they wanted access to rapid wastewater data even if information available to determine public health risk or which actions should be taken is insufficient. Wastewater data can help keep the public informed and should be accompanied by clear public health interpretations.

## Footnotes

* These authors contributed equally to this report.

†
www.cdc.gov/wastewater/

§
https://www.porternovelli.com/

¶ 45 C.F.R. part 46, 21 C.F.R. part 56; 42 U.S.C. Sect. 241(d); 5 U.S.C. Sect. 552a; 44 U.S.C. Sect. 3501 et seq.

**
https://www.census.gov/programs-surveys/cps.html

## References

[1] Adams C, Bias M, Welsh RM, The National Wastewater Surveillance System (NWSS): from inception to widespread coverage, 2020–2022, United States. Sci Total Environ 2024;924:171566. 10.1016/j.scitotenv.2024.171566 38461979 PMC11103741

[2] Turner H, Horter L, Welton M, Qualitative assessment of a novel results-based partnership between national wastewater surveillance centers of excellence and utility companies, Houston (Texas), Colorado, Wisconsin, and California, 2023. Research Square [Preprint posted online August 21, 2024]. 10.21203/rs.3.rs-4796194/v1 PMC13101207 41832489

[3] The Lancet Microbe. Wastewater: between surveillance and intrusion. Lancet Microbe 2024;5:e509. 10.1016/S2666-5247(24)00132-0 38797191

[4] Graeff TR. Response bias. In: Kempf-Leonard K, ed. Encyclopedia of social measurement. 1st ed. New York, NY: Elsevier; 2005:411–8.

